# Extensile lateral versus sinus tarsi approach for displaced, intra-articular calcaneal fractures: a meta-analysis

**DOI:** 10.1186/s13018-018-0943-6

**Published:** 2018-09-24

**Authors:** Cyrus Rashid Mehta, Vincent V. G. An, Kevin Phan, Brahman Sivakumar, Andrew J. Kanawati, Mayuran Suthersan

**Affiliations:** Orthopaedics Department, Westmead Hospital, Corner of Hawkesbury and Darcy Roads, Westmead, Sydney, Australia

**Keywords:** Calcaneus, Intra-articular fracture, Extensile lateral approach, Sinus tarsi approach, Minimally invasive

## Abstract

**Background:**

Operative management of displaced, intra-articular calcaneal fractures is associated with improved functional outcomes but associated with frequent complications due to poor soft tissue healing. The use of a minimally invasive sinus tarsi approach to the fixation of these fractures may be associated with a lower rate of complications and therefore provide superior outcomes without the associated morbidity of operative intervention.

**Methods:**

We reviewed four prospective and seven retrospective trials that compared the outcomes from the operative fixation of displaced intra-articular calcaneal fractures via either an extensile lateral approach or minimally invasive fixation via a sinus tarsi approach.

**Results:**

Patients managed with a sinus tarsi approach were less likely to suffer complications (OR = 2.98, 95% CI = 1.62–5.49, *p* = 0.0005) and had a shorter duration of surgery (OR = 44.29, 95% CI = 2.94–85.64, *p* = 0.04).

**Conclusion:**

In displaced intra-articular calcaneal fractures, a minimally invasive sinus tarsi approach is associated with a lower complication rate and quicker operation duration compared to open reduction and internal fixation via an extensile lateral approach.

## Introduction

Calcaneal fractures account for approximately 1–2% of all fractures of the human body, with an annual incidence of 11.5 per 100,000 people. Displaced intra-articular fractures comprise 60–75% of calcaneal fractures [1, 2]. Conservative management of these injuries is often sub-optimal, resulting in arthritis of the subtalar joint, malunion and poor functional outcomes [3]. In appropriately selected patients, operative fixation is therefore favoured in managing displaced intra-articular fractures of the calcaneus [4, 5]. The traditional approach to fixation has been open reduction and internal fixation (ORIF) through an extensile L-shaped lateral approach (ELA) [6]. The extensile lateral approach has traditionally been utilized for the fixation of most displaced intraarticular calcaneal fractures. The skin incision is L-shaped with the horizontal limb in line with the fifth metatarsal and the vertical limb is between the Achilles tendon and fibula. The incision is carried directly to the bone in order to create thick soft tissue flaps. Proximal extension of the flap allows exposure of the subtalar joint. The primary danger with this approach is damage to the blood supply to the corner of the L-shaped flap. This area receives its blood supply from the lateral calcaneal artery [7]. The use of this approach is complicated by a relatively high risk of wound infection and breakdown [8–12]. Minimally invasive reduction and fixation techniques via a sinus tarsi approach (STA) have been developed in an attempt to avoid the potential complications associated with an extensile lateral approach [13–16]. It utilizes a small incision that is based distal to the fibula and anterior to the peroneal tendons. The smaller incision has a lower theoretical risk of damage to the sural nerve and the lateral calcaneal artery which are at risk during an extensile lateral approach. Following dissection through subcutaneous fat and fascia, the subtalar joint is identified and a small capsulotomy allows excellent visualization of the articular surface to assess reduction. Wound failure, breakdown, or infection can have devastating consequences and is extremely difficult to deal with. Any means by which these complications can be reduced should be investigated and utilized if they are proven to be effective.

## Materials and methods

In December 2016, a search was conducted on the PubMed and MEDLINE databases using the keywords displaced intra-articular calcaneal fracture, open reduction and internal fixation, sinus tarsi approach, extensile lateral approach, minimally invasive and percutaneous. The references of the articles found were also reviewed to identify additional studies for inclusion. Studies were included in the meta-analysis if they met the following criteria: (1) sample population at skeletal maturity, (2) sample size > 1 (i.e. not a case study) and (3) investigated outcome measures (both quantitative and qualitative) between ORIF and minimally invasive fixation. Studies that included patients with bilateral or concurrent injuries secondary to trauma were not excluded from the meta-analysis due to the high rate of associated injuries with calcaneal fractures (up to 50%) and bilateral fractures (5–10%) in the general population [1].

Data were extracted by two independent reviewers with any disagreement resolved by consultation of a third reviewer. Outcome variables that were assessed included wound and neurovascular complications, rate of reoperation, operating time, time to surgery and postoperative articular displacement.

The relative risk (RR) was used as a summary statistic for dichotomous variables and weighted mean difference (WMD) for continuous variables. In the present study, both fixed and random effect models were tested. In the fixed effects model, it was assumed that the treatment effect in each study was the same, whereas in a random-effects model, it was assumed that there were variations between studies. χ^2^ tests were used to study heterogeneity between trials. *I*^2^ statistic was used to estimate the percentage of total variation across studies, owing to heterogeneity rather than chance, with values greater than 50% considered as substantial heterogeneity. *I*^2^ can be calculated as *I*^2^ = 100% × (*Q* − df)/*Q*, with *Q* defined as Cochrane’s heterogeneity statistics and df defined as the degree of freedom [17]. The fixed effects model was presented when there was insignificant heterogeneity as defined by *I*^2^ < 50% and *P* < 0.05, whereas the random effects model was used when heterogeneity was deemed significantly with *I*^2^ ≥ 50% and *P* < 0.05 for heterogeneity [18]. Specific analyses considering confounding factors were not possible because raw data were not available. All *P* values were two-sided. All statistical analysis was conducted with Review Manager Version 5.3.2 (Cochrane Collaboration, Software Update, Oxford, UK).

## Results

**Fig. 1:**
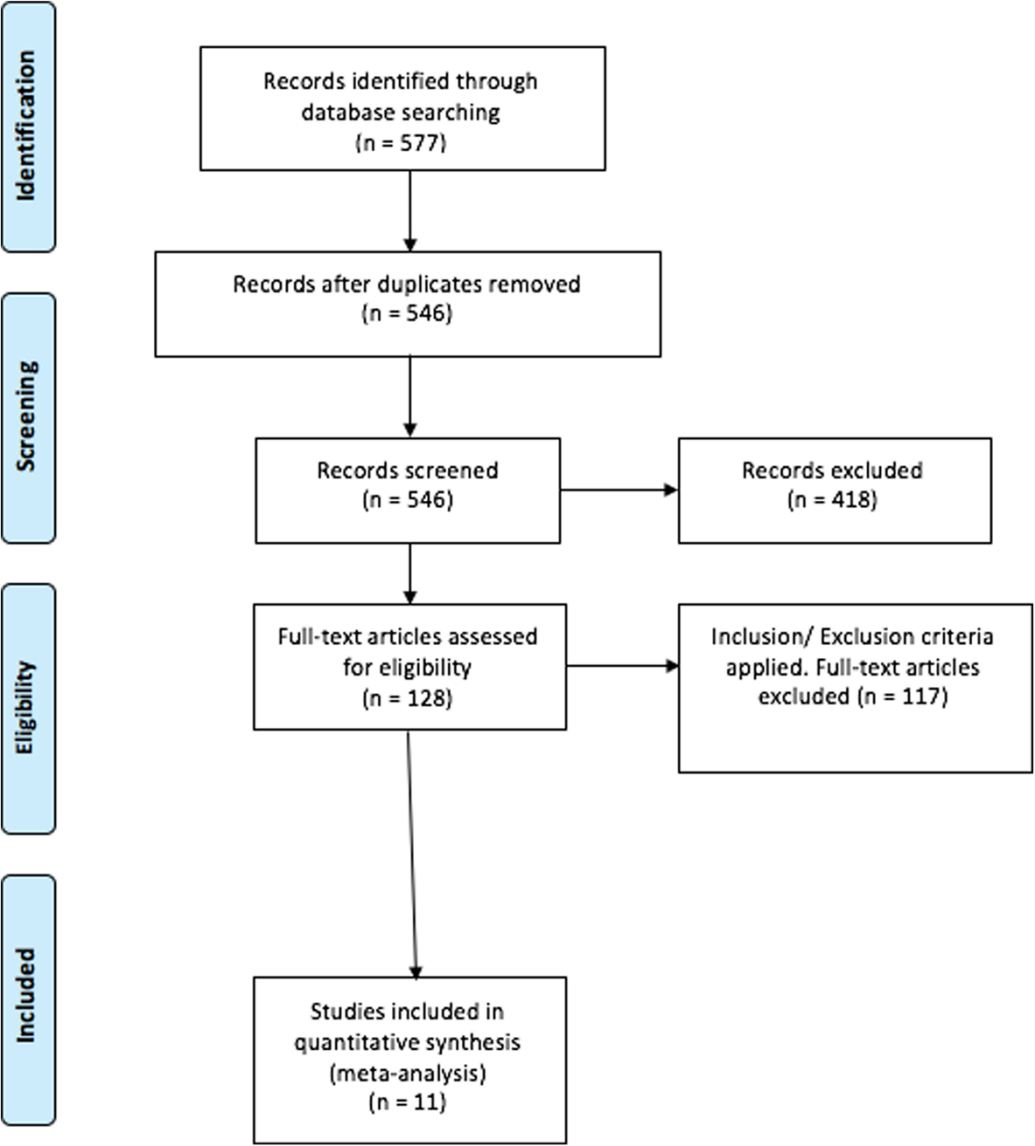
PRISMA flow diagram

Five hundred and seventy seven studies were reviewed, of which 11 were identified that met the above criteria (Fig. 1). All studies were published from 2008 to 2016. Seven of the studies were retrospective analyses of data, with the remaining being prospective randomised trials. The final sample comprised 1131 patients in total, of whom 557 underwent ORIF via a lateral approach and 574 underwent percutaneous fixation. With bilateral injuries accounted for, there were 594 fractures in the ORIF and 622 in the percutaneous fixation group. The average age of participants was reported in 10 of the 11 studies and ranged between 30 and 46 years (Table 1).

**Table 1:** Study characteristics

Study	Type of study	ELA vs. STA		
		Average age (years)	No. of patients	No. of fractures
Takasaka 2016 [22]	Retrospective	Not specified	20 vs. 27	23 vs. 27
Kumar 2014 [23]	Prospective	30 vs. 31	21 vs. 21	23 vs. 22
Chen 2011 [24]	Prospective	32 vs. 31	40 vs. 38	40 vs. 38
Wang 2015 [25]	Retrospective	41 vs. 39	53 vs. 54	58 vs. 60
DeWall 2010 [26]	Retrospective	41 vs. 40	41 vs. 79	42 vs. 83
Basile 2016 [27]	Prospective	39 vs. 41	20 vs. 18	20 vs. 18
Kline 2013 [28]	Retrospective	42 vs. 46	79 vs. 33	79 vs. 33
Yeo 2015 [29]	Retrospective	42 vs. 46	60 vs. 40	60 vs. 40
Xia 2014 [30]	Prospective	37 vs. 38	49 vs. 59	53 vs. 64
Wu 2012 [31]	Retrospective	41 vs. 39	148 vs. 181	170 vs. 213
Weber 2008 [32]	Retrospective	40 vs. 42	26 vs. 24	26 vs. 24

**Fig. 2:**
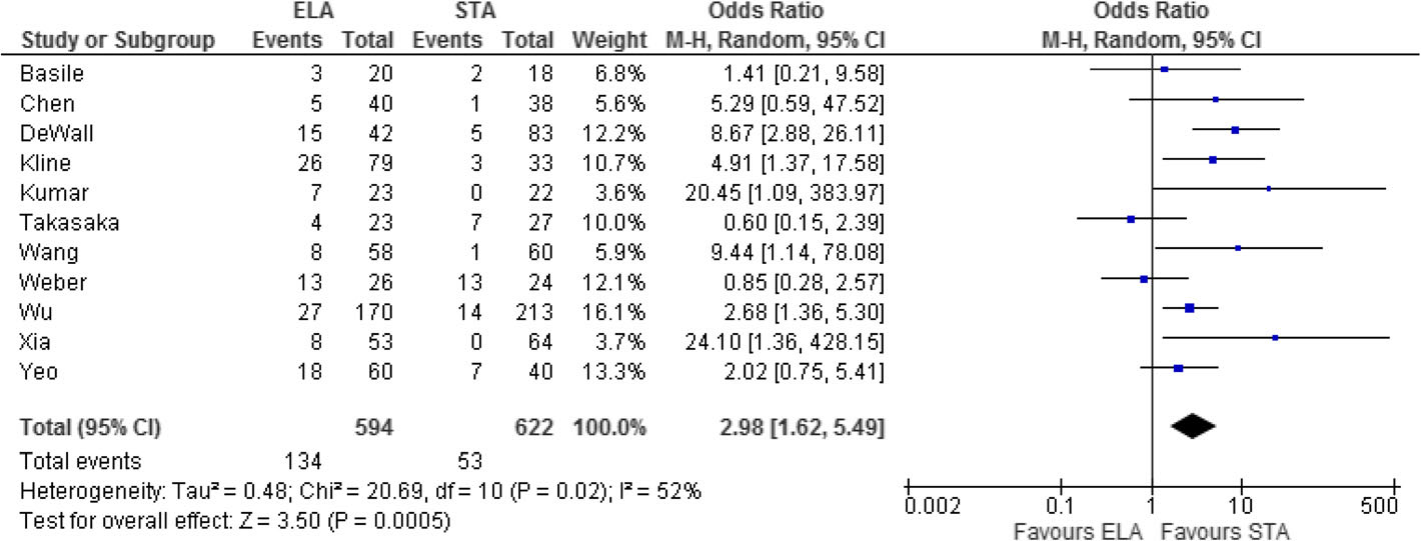
Forest plot for postoperative complications

All studies investigated the occurrence of complications postoperatively (Table 2). Patients who underwent ELA were more likely to suffer postoperative complications (OR = 2.98, 95% CI = 1.62–5.49, *p* = 0.0005, Fig. 2).

**Table 2:** Complications

Study	Complications	
	ELA	STA
Takasaka 2016 [22]	4 (1 infection, 2 skin necrosis, and 1 sural nerve neuroma)	0
Kumar 2014 [23]	7 (3 wound dehiscence, 1 superficial infection, and 3 deep infections)	0
Chen 2011 [24]	5 (2 deep infections and 3 superficial wound infection)	1 superficial wound infection
Wang 2015 [25]	8 (2 deep infections and 6 poor wound healing)	1 pin site ooze
DeWall 2010 [26]	15 (9 minor wound complications and 6 deep infections)	5 minor wound complications
Basile 2016 [27]	3 (2 wound edge necrosis and 1 wound breakdown requiring skin flap)	2 (1 mal-reduction and 1 tendon irritation requiring re-operation)
Kline 2013 [28]	26 (23 wound healing and 3 sural neuropathy)	3 (2 wound healing and 1 sural nerve neuropathy)
Wu 2012 [31]	27 (12 superficial infections, 6 wound edge necrosis, 2 deep infections, 7 sural nerve neuropathy, and 4 defects with plate removal)	14 (4 superficial infections, 3 sural nerve injuries, 7 medial injuries specific to this technique, and 4 defects with plate removal)
Xia 2014 [30]	8 (6 dehiscence/superficial infection and 2 wound edge necrosis)	0
Yeo 2015 [29]	18 (8 wound complications, 4 sural nerve injury, 1 peroneal tendonitis, and 5 subtalar stiffness)	7 (2 wound complications, 2 sural nerve injury, and 3 subtalar stiffness)
Weber 2008 [32]	13 (1 delayed wound healing, 1 hematoma, 1 sural nerve injury, 4 complex regional pain syndrome, 3 hardware removals, and 3 subsequent subtalar arthrodeses)	11 (1 plantar nerve irritation, 10 scar tenderness at 3 months post-op requiring hardware removal)

**Fig. 3:**
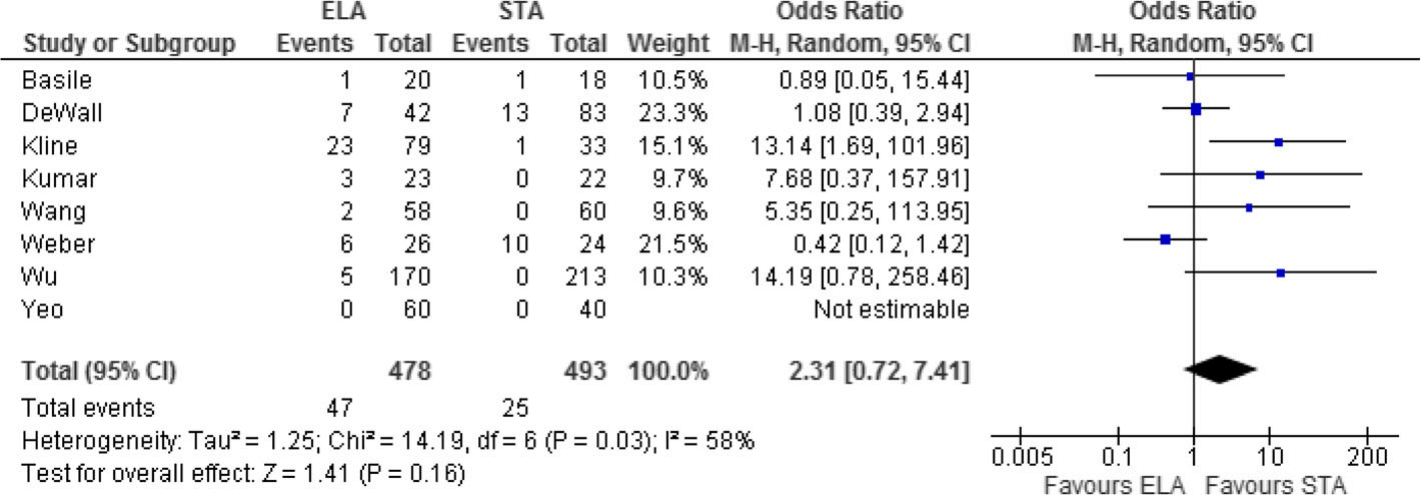
Forest plot for reoperation rate

**Fig. 4:**
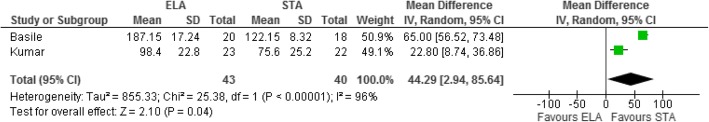
Forest plot for operation duration

**Fig. 5:**
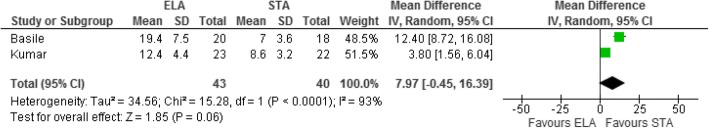
Forest plot for the time from injury to surgery

**Fig. 6:**
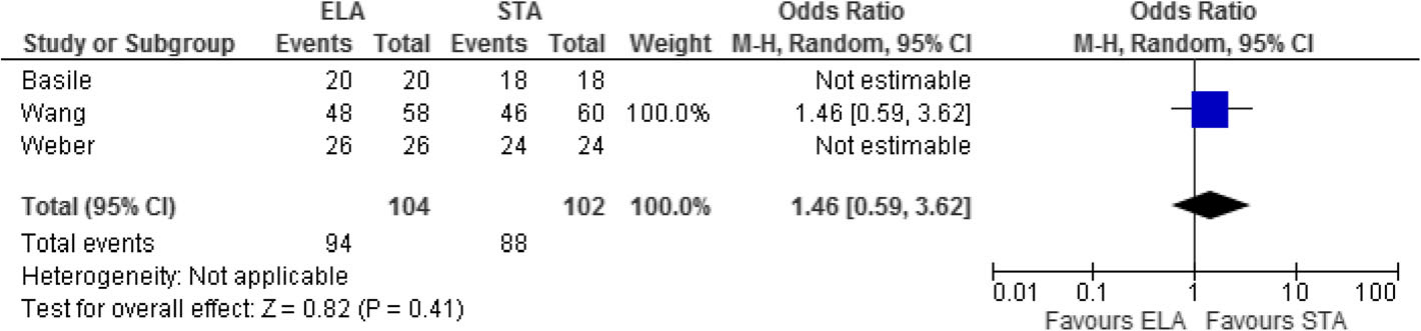
Forest plot for postoperative articular displacement

Eight studies investigated reoperation rates. Patients in the ELA group were more likely to have reoperations; however, this difference was not statistically significant (mean difference = 2.31, 95% CI = 0.72–7.41, *p* = 0.16, Fig. 3). STA was associated with statistically significant shorter operating times in two studies that compared the outcome (OR = 44.29, 95% CI = 2.94–85.64, *p* = 0.04, Fig. 4). The time taken from injury to surgery was also assessed by these two studies and suggested a faster time to operation for those in the STA group; however, this was not statistically significant (mean difference = 7.97, 95% CI = − 0.45–16.39, *p* = 0.06 Fig. 5). Three studies assessed postoperative articular displacement and suggested a favourable outcome after STA, but this result was not significant (OR = 1.46, 95% CI = 0.59–3.62, *p* = 0.41, Fig. 6).

Additional data and outcome variables (e.g. postoperative Bohler’s angle, AOFAS score) were also measured by the studies that were unable to be included in a meta-analysis due to a lack of reported standard deviations for the values or the outcomes being assessed by only a single study, precluding formal meta-analysis.

## Discussion

A review of the literature reveals a paucity of valid, objective data determining the difference between ELA and STA. Bai et al. conducted a meta-analysis of four randomised controlled trials and three cohort studies. Their study attempted to show a reduction in the complication rate and operating time; however, the inclusion of primarily cohort studies in the meta-analysis mandates caution when interpreting their results. Additionally, the total number of patients included in their meta-analysis was only 532 [19]. Zeng et al. similarly completed a meta-analysis of minimally invasive versus extensile lateral approaches for Sanders type 2 and 3 calcaneal fractures but were only able to include 495 participants from eight randomised trials [20]. Lastly, Yao et al. report a meta-analysis of 1078 participants on the topic and claim to have identified improved wound healing and functional outcomes associated with a sinus tarsi approach. Whilst promising, their meta-analysis included participants sourced from only two randomised controlled trials, with the majority of participants being from case series [21]. The results are therefore not as valid as those in the present study, due to the inherent bias associated with conducting and making conclusions based on cohort studies and case series.

These sample sizes are also substantially smaller than our population of 1131, the majority of which were sourced from randomised controlled trials which increases the validity of the present study when compared to previously published results.

From our meta-analysis, a minimally invasive sinus tarsi approach for the fixation of displaced intra-articular calcaneal fractures is associated with a lower rate of complications and a faster operation time when compared to ORIF via an extensile lateral approach. Soft tissue complications including deep and superficial wound infections, sural nerve damage and skin necrosis are more common in patients managed with ELA.

A major limitation of the current evidence comparing the two approaches is the lack of data on functional outcomes and post-operative articular displacement.

Seven of the studies in our meta-analysis assessed the functional outcomes (via the AOFAS) between the two approaches. However, these results were unable to be included in a meta-analysis as they were not accompanied by standard deviations. Therefore, interpretation of the raw data would lead to inaccurate conclusions regarding the patients’ functional outcomes. Further studies should therefore look at assessing the function post-operatively in a way that allows a statistical analysis of a larger sample size of patients.

Similarly, three studies (Bastille, Weber, Wang) assessed post-operative residual articular displacement based on CT scans. In spite of conventional teaching that an ELA provides improved visualization of the fracture compared to STA, there was a general trend towards less articular displacement when the STA was utilized; these results were not statistically significant (Fig. 5). Given that one of the main goals of fixation is an anatomic articular reduction which will inevitably influence post-operative function, more research is definitely required in order to determine which of the two procedures yields less articular displacement. A randomised controlled trial utilizing post-operative CT scans would be very useful in studying this and should be the direction of future research on this topic.

This was a very extensive meta-analysis; however, the main limitation was the lack of uniformity of outcome reporting between the randomised trials.

## Conclusion

In displaced intra-articular calcaneal fractures, a minimally invasive sinus tarsi approach is associated with a lower complication rate and quicker operation duration compared to open reduction and internal fixation via an extensile lateral approach.

## Abbreviations

AOFAS: American Orthopedic Foot and Ankle Society Score
CI: Confidence interval
ELA: Extensile lateral approach
OR: Odds ratio
ORIF: Open reduction and internal fixation
RR: Relative risk
STA: Sinus tarsi approach
WMD: Weighted mean difference
